# Distribution of laminin and fibronectin isoforms in oral mucosa and oral squamous cell carcinoma

**DOI:** 10.1038/sj.bjc.6690809

**Published:** 1999-11

**Authors:** H Kosmehl, A Berndt, S Strassburger, L Borsi, P Rousselle, U Mandel, P Hyckel, L Zardi, D Katenkamp

**Affiliations:** Institute of Pathology andClinic of Maxillofacial Surgery, Friedrich Schiller University, Jena D-07740, Germany; Centro di Biotecnologie, Genova, Italy; Institute de Biologie et Chimie des Proteins, Universite Claude Bernard Lyon I, Lyon, France; Faculty of the Health Sciences, School of Dentistry, University of Copenhagen, Copenhagen, Denmark

**Keywords:** oral squamous cell carcinoma, laminin isoforms, fibronectin isoforms, ED-B fibronectin in situ hybridization, myofibroblasts, oncofetal extracellular matrix

## Abstract

The expression of laminin and fibronectin isoforms varies with cellular maturation and differentiation and these differences may well influence cellular processes such as adhesion and motility. The basement membrane (BM) of fetal oral squamous epithelium contains the laminin chains, α2, α3, α5, β1, β2, β3, γ1 and γ2. The BM of adult normal oral squamous epithelium comprises the laminin chains, α3, α5, β1, β3, γ1 and γ2. A re-expression of the laminin α2 and β2 chains could be shown in adult hyperproliferative, dysplastic and carcinomatous lesions. In dysplasia and oral squamous cell carcinoma (OSCC), multifocal breaks of the BM are present as indicated by laminin chain antibodies. These breaks correlate to malignancy grade in their extent. Moreover, in the invasion front the α3 and γ2 chain of laminin-5 can immunohistochemically be found outside the BM within the cytoplasm of budding carcinoma cells and in the adjacent stroma. The correlation between the morphological pattern of invasive tumour clusters and a laminin-5 immunostaining in the adjacent stroma may suggest, first, that a laminin-5 deposition outside the BM is an immunohistochemical marker for invasion and second, that OSCC invasion is guided by the laminin-5 matrix. Expression of oncofetal fibronectins (IIICS de novo glycosylated fibronectin and ED-B fibronectin) could be demonstrated throughout the stromal compartment. However, the ED-B fibronectin synthesizing cells (RNA/RNA in situ hybridization) are confined to small stroma areas and to single stroma and inflammatory cells in the invasion front. A correlation of the number of ED-B fibronectin synthesizing cells to malignancy grade could not be seen. ED-B fibronectin mRNA-positive cells seem to be concentrated in areas of fibrous stroma recruitment with a linear alignment of stromal fibro-/myofibroblasts (desmoplasia). Double staining experiments (ED-B fibronectin in situ hybridization and α-smooth muscle actin immunohistochemistry) indicated that the stroma myofibroblasts are a preferential source of ED-B fibronectin. In conclusion, in OSCC, a fetal extracellular matrix conversion is demonstrable. Tumour cells (laminin α2 and β2 chain) and recruited stromal myofibroblasts (oncofetal ED-B fibronectin) contribute to the fetal extracellular matrix milieu. © 1999 Cancer Research Campaign


*British Journal of Cancer* (1999) **81**(6), 1071–1079
© 1999 Cancer Research Campaign
Article no. bjoc.1999.0809

### Distribution of laminin and fibronectin isoforms in oral mucosa and oral squamous cell carcinoma

**H Kosmehl[1], A Berndt[1], S Strassburger[2], L Borsi[3], P Rousselle[4], U Mandel[5], P Hyckel[2], L Zardi[3] and D Katenkamp[1]**

[1]Institute of Pathology and [2]Clinic of Maxillofacial Surgery, Friedrich Schiller University, D-07740 Jena, Germany; [3]Centro di Biotecnologie, Genova, Italy; [4]Institute de Biologie et Chimie des Proteins, Universite Claude Bernard Lyon I, Lyon, France; [5]Faculty of the Health Sciences, School of Dentistry, University of Copenhagen, Copenhagen, Denmark

**Summary** The expression of laminin and fibronectin isoforms varies with cellular maturation and differentiation and these differences may well influence cellular processes such as adhesion and motility. The basement membrane (BM) of fetal oral squamous epithelium contains the laminin chains, α2, α3, α5, β1, β2, β3, γ1 and γ2. The BM of adult normal oral squamous epithelium comprises the laminin chains, α3, α5, β1, β3, γ1 and γ2. A re-expression of the laminin α2 and β2 chains could be shown in adult hyperproliferative, dysplastic and carcinomatous lesions. In dysplasia and oral squamous cell carcinoma (OSCC), multifocal breaks of the BM are present as indicated by laminin chain antibodies. These breaks correlate to malignancy grade in their extent. Moreover, in the invasion front the α3 and γ2 chain of laminin-5 can immunohistochemically be found outside the BM within the cytoplasm of budding carcinoma cells and in the adjacent stroma. The correlation between the morphological pattern of invasive tumour clusters and a laminin-5 immunostaining in the adjacent stroma may suggest, first, that a laminin-5 deposition outside the BM is an immunohistochemical marker for invasion and second, that OSCC invasion is guided by the laminin-5 matrix. Expression of oncofetal fibronectins (IIICS de novo glycosylated fibronectin and ED-B fibronectin) could be demonstrated throughout the stromal compartment. However, the ED-B fibronectin synthesizing cells (RNA/RNA in situ hybridization) are confined to small stroma areas and to single stroma and inflammatory cells in the invasion front. A correlation of the number of ED-B fibronectin synthesizing cells to malignancy grade could not be seen. ED-B fibronectin mRNA-positive cells seem to be concentrated in areas of fibrous stroma recruitment with a linear alignment of stromal fibro-/myofibroblasts (desmoplasia). Double staining experiments (ED-B fibronectin in situ hybridization and α-smooth muscle actin immunohistochemistry) indicated that the stroma myofibroblasts are a preferential source of ED-B fibronectin. In conclusion, in OSCC, a fetal extracellular matrix conversion is demonstrable. Tumour cells (laminin α2 and β2 chain) and recruited stromal myofibroblasts (oncofetal ED-B fibronectin) contribute to the fetal extracellular matrix milieu. © 1999 Cancer Research Campaign

**Keywords:** oral squamous cell carcinoma; laminin isoforms; fibronectin isoforms; ED-B fibronectin in situ hybridization; myofibroblasts; oncofetal extracellular matrix

In oral squamous cell carcinoma (OSCC), the cellular differentiation in the invasion front as well as the mode of invasion are crucial for the tumour behaviour. These parameters belong to the so-called *new malignancy grading*, which yields a better prognostic value than the conventional Broder's grading system (Bryne et al, 1992). Therefore, an analysis of the extracellular matrix in the invasion front of OSCC may improve the understanding of tumour cell–matrix interactions during malignant growth.

The epithelium–stroma interface is delineated by a distinct extracellular matrix structure, the basement membrane (BM). Structural irregularities of carcinoma BMs, including the loss of BM material, have been known for some time. In OSCC the extent of BM defects correlates with invasive and metastatic potential (Harada et al, 1994; Kobayashi et al, 1995). In areas with BM defects the invading carcinoma cells may come into contact with the abundant stromal fibronectin matrix.

Recent results have given rise to a new definition of laminin and fibronectin as molecule families (Mandel et al, 1994; Kosmehl et al, 1996). Laminin is a heterotrimeric molecule with an α, β and γ chain. Laminin isoforms arise from an exchange of single chains and can be identified in situ by chain-specific antibodies. Laminin isoforms are deposited in a maturation and differentiation-dependent manner. Present knowledge of the laminin chain pattern in normal and diseased oral squamous epithelium is only incomplete.

Whilst laminin is quantitatively the most important non-collageneous matrix protein of the BM, fibronectin is nearly ubiquitously distributed. Fibronectin is a high-molecular-mass glycoprotein with a molecular polymorphism generated by alternative processing of the pre-mRNA (alternative splicing) or by post-translational modifications of the protein itself (de novo glycosylation). Soluble plasma fibronectin (dimeric, alternatively spliced in the IIICS region) is synthesized by hepatocytes and is found in body fluids. Cellular fibronectin is a larger molecule with an additional domain in the IIICS region and is constituent of the extracellular matrix in dimeric or multimeric cross-linked form. Variants of cellular fibronectin arise from further alternative splicing or de novo O-linked glycosylation in the IIICS domain. In contrast to the ubiquitously distributed common cellular fibronectin, the ED-A and the ED-B fibronectin as well as the de novo glycosylated fibronectin isoforms are limited to areas with structural tissue modulation, embryonic tissues, reparative processes and neoplasias (Loridon-Rosa et al, 1990; Xia and Culp,

*Received 26 June 1998*
*Revised 30 March 1999*
*Accepted 30 March 1999*

*Correspondence to:* H Kosmehl

1995; Kornblihtt et al, 1996; Kosmehl et al, 1996; Burton-Wurster et al, 1997). The isoforms are identifiable using specific monoclonal antibodies (Borsi et al, 1987; Mandel et al, 1992; Carnemolla et al, 1996).

Extracellular matrix molecules influence differentiation, proliferation, migration, and have stabilizing and separating functions (Kosmehl et al, 1996). To our knowledge, a comparative study focusing on the modulation of the laminin chain pattern in fetal, normal adult, hyperproliferative, or dysplastic oral epithelium and in OSCC of different malignancy grade has not yet been conducted. Although an immunohistochemical study on the expression of so-called oncofetal fibronectins (ED-B and de novo glycosylated fibronectin) in relation to the formation of an invasive phenotype of neoplastic oral epithelium has already been published (Mandel et al, 1994), the source of the ED-B fibronectin within the carcinoma remains unclear.

### MATERIALS AND METHODS

### Tissue material

Twenty-seven surgical specimens of squamous cell carcinoma of the oral cavity (tumour invasion front), five samples of normal adult non-neoplastic, two samples of normal fetal (5th–6th month of gestation), five samples of hyperplastic and three samples of dysplastic oral squamous epithelium were available for histological and immunohistochemical investigations. All samples are derived from the Clinic of Maxillofacial Surgery of the Friedrich Schiller University of Jena. Blocks of fresh tissue measuring 4 × 4 × 4 mm were immediately shock-frozen in isopropanol, cooled by liquid nitrogen and stored at –75°C. The diagnosis was made on conventional haematoxylin and eosin (H&E)-stained serial sections and confirmed in the corresponding paraffin-embedded tissue. Histological grading of malignancy was performed using the method reported by Bryne et al (1992). For correlation analysis the tumours were grouped into well-differentiated G1 carcinomas and into moderate to poorly differentiated G2/3 carcinomas.

### Immunohistochemistry

Cryostat sections of the respective frozen tissue samples were fixed in ice-cooled acetone for 15 min and subjected to immunohistochemistry. Primary antibodies used are listed in Table 1. The immunostaining for collagen type IV was used as reference for the BM. The laminin chain immunostainings were interpreted behind this background.

Immunohistochemical staining was performed using the alkaline phosphatase monoclonal anti-alkaline phosphatase (APAAP) method. The primary antibody was incubated for 30 min at room temperature. After washing with Tris buffer, sections were treated with rabbit anti-mouse immunoglobulin (diluted 1:70, Dako, Denmark), and then with the mouse APAAP complex (Dako, Denmark). Both incubations were carried out for 30 min at room temperature. To increase the staining intensity, the incubation with the rabbit anti-mouse immunoglobulin and with the APAAP complex was repeated twice. Naphthol-AS-biphosphate (Sigma, USA) and new fuchsin (Merck, Germany) were used as substrate and developer respectively. To inhibit endogenous tissue enzyme activity, the developing solution was supplemented with 0.25 mmol $l^{-1}$ levamisole (Sigma, USA). As negative control, the primary antibody was replaced by non-immune serum.

**Table 1** Primary antibodies and their reactive determinants

| Antigen | Clone | Dilution | References |
|---|---|---|---|
| Lam α1 chain | goat polyclonal | 1:100 | Santa Cruz Biotechnology, Inc. (sc 6016) |
| Lam α2 chain | 5H2 | 1:5000 | Engvall et al, 1990 |
| Lam α3 chain | BM165 | 1:5000 | Rousselle et al, 1991 |
| | P3E4 (epiligrin) | 1:100 | Wayner et al, 1993 |
| Lam α5 chain | 4C7 | 1:10 000 | Tiger et al, 1997 |
| Lam β1 chain | 4E10 | 1:20 000 | Wewer et al, 1983 |
| Lam β2 chain | C4 | 1:3000 | Sanes and Chiu, 1983 |
| Lam β3 chain | 6F12 | 1:50 | Marinkovich et al, 1992 |
| Lam γ1 chain | 2E8 | 1:500 | Wewer et al, 1983 |
| Lam γ2 chain | GB3 | 1:20 | Hsi and Yeh, 1986 |
| Collagen type IV | CIV22 | 1:50 | Odermatt et al, 1984 |
| Fibronectin (all variants) | IST4 | 1:20 | Zardi et al, 1980 |
| ED-B$^{+}$ fibronectin | BC-1 | 1:20 | Carnemolla et al, 1989 |
| | CGS1 (recombinant antibody) | 1:50 | Carnemolla et al, 1996 |
| De novo glycosylated fn | 5C10 | 1:20 | Mandel et al, 1992 |
| | FDC-6 | 1:10–1:20 | Matsuura and Hakamori, 1985 |
| Von Willebrand factor | F8/86 | 1:50 | Hruban et al, 1887 |
| α smooth muscle actin | 1A4 | 1:50 | Skalli et al, 1986 |

### Quantitative evaluation of the laminin chain and collagen type IV immunostainings and statistical analysis

The BM defects for the laminin chains α3, α5, β1, γ1 and collagen type IV were semiquantitatively evaluated in 30 tumour cell clusters per case at the invasion front according to the criteria introduced by Kobayashi et al (1995) and scored as follows:

- continuous linear staining (no BM defects, score 0)
- loss of staining in less than 10% of the tumour–stromal interface per tumour cell nest (minor BM defects, score 1)
- loss of staining in less than 50% of the tumour–stromal interface per tumour cell nest (moderate BM defects, score 2)
- loss of staining in more than 50% of the tumour–stromal interface per tumour cell nest (BM defects to a large extent, score 3).

The number of tumour cell clusters with minor, moderate and major BM defects are presented as mean values ± standard deviation (s.d.).

For statistical evaluation of laminin chain and collagen type IV defects in relation to malignancy grade (G1 and G2/3) the products of the score and number of related tumour cell clusters were added up for each tumour. Statistical analysis was done using the Mann–Whitney *U*-test (SPSS, Microsoft). $P < 0.05$ was regarded as statistically significant.

### In situ hybridization

The cDNA fragment of the ED-B portion of human fibronectin was inserted as a 272 bp *Bam*HI fragment into *Bam*HI digest pBluescriptIIKS+ vector (Stratagene, La Jolla, CA, USA). Sense or antisense RNA probes were generated by in vitro transcription of the *Xba*I or *Eco*RI linearized plasmid using digoxigenin-labelled uridine triphosphate as substrate (DIG RNA Labelling

© 1999 Cancer Research Campaign

**Table 2** Extent of BM defects in the invasion front of oral squamous cell carcinomas in correlation to malignancy grade. The mean value (+/– standard deviation) of the number of tumour nests with no, minor, moderate or large extended BM defects of all G1 and G2/3 tumours is presented evaluating 30 tumour cell nests per case

| Laminin chain | No BM defects | | Minor BM defects | | Moderate BM defects | | BM defects to a large extent | |
|---|---|---|---|---|---|---|---|---|
| | G1 | G2/3 | G1 | G2/3 | G1 | G2/3 | G1 | G2/3 |
| α3 | 18.8 (± 8.85) | 5.0 (± 7.36) | 5.8 (± 4.02) | 7.3 (± 4.60) | 3.4 (± 3.34) | 8.8 (± 4.25) | 2.0 (± 3.23) | 8.9 (± 7.92) |
| α5 | 14.6 (± 9.74) | 1.6 (± 2.75) | 6.5 (± 3.24) | 5.1 (± 4.74) | 4.7 (± 3.74) | 8.4 (± 4.38) | 4.2 (± 6.81) | 14.9 (± 9.71) |
| β1 | 13.0 (± 9.25) | 2.9 (± 4.93) | 8.0 (± 4.27) | 5.1 (± 4.03) | 5.0 (± 3.43) | 8.6 (± 3.50) | 4.0 (± 6.70) | 13.3 (± 8.06) |
| γ1 | 5.9 (± 7.82) | 0.4 (± 0.81) | 5.9 (± 5.06) | 2.6 (± 3.61) | 7.2 (± 3.85) | 8.0 (± 4.18) | 11.0 (± 9.52) | 19.0 (± 7.63) |
| Collagen type IV | 12.5 (± 8.83) | 2.9 (± 3.82) | 7.2 (± 3.93) | 6.1 (± 4.40) | 5.4 (± 2.91) | 8.8 (± 3.67) | 4.9 (± 5.53) | 12.2 (± 9.23) |

Kit, Boehringer Mannheim, Germany) and T3 or T7 RNA polymerase. Unincorporated nucleotides were removed by ethanol precipitation. The precipitate was dissolved in 100-μl diethylpyrocarbonate treated and RNAase inhibitor containing water. The transcripts were analysed by agarose gel electrophoresis and dot blot assay.

In situ hybridization was carried out on 7 to 10-μm-thick sections of immediately snap-frozen tissue. Sections were heated for 2 min at 50°C to fix the RNA in the tissue. The sections were allowed to air dry for 30 min and then fixed in 4% (w/v) paraformaldehyde in phosphate-buffered saline (PBS) for 20 min at 4°C. After washing in PBS (1 × 5 min) and 2 × standard saline citrate (SSC) (2 × 5 min), the prehybridization solution was applied on each slide for 60 min at 37°C. The prehybridization solution contained 4 × SSC, 10% dextran sulphate, 1 × Denhardt's solution, 2 mM EDTA, 50% de-ionized formamide, 100 μg $ml^{-1}$ herring sperm DNA and 100 μg $ml^{-1}$ tRNA. After removal of this solution, each section was covered with 100 μl of prehybridization solution containing 200–1000 ng $ml^{-1}$ of DIG-labelled antisense cRNA probe and incubated at 37°C overnight.

As hybridization controls, the antisense cRNA probe was replaced by ED-B sense cRNA or was completely omitted from the hybridization to evaluate the quality of the colour detection system. After hybridization, unbound probe was washed off from the sections as follows: 1 × 5 min with 2 × SSC at 37°C, 3 × 5 min with 60% formamide in 0.2 × SSC at 37°C, and 2 × 5 min with 2 × SSC at room temperature.

Hybridized DIG-labelled cRNA probes were detected using the components of the DIG Nucleic Acid Detection Kit (Boehringer Mannheim, Germany) following the manufacturer's recommended protocol. Colour reaction was carried out for up to 24 h at room temperature.

The ED-B fibronectin mRNA is situ hybridization and the corresponding immunohistochemistry using the monoclonal antibody BC1 and the recombinant antibody CGS1 was performed on 13 OSCC. Four carcinomas were well-differentiated (G1), and nine carcinomas were moderately to poorly differentiated (six G2 and three G3). The number of in situ hybridization signal bearing cells was counted in 10 HPF (× 40) and was correlated to tumour grade (G1, G2/3). To prove statistical significance, the Kruskal–Wallis and Mann–Whitney *U*-tests were applied (SPSS, Microsoft).

### Double staining procedure for α-smooth muscle actin/Von Willebrand factor and ED-B fibronectin synthesis

A combination of in situ hybridization and PAP technique was used for double staining of cells showing ED-B fibronectin synthesis and positivity for alpha-smooth muscle actin or Von Willebrand factor. First, the mRNA in situ hybridization for ED-B fibronectin was performed as described. Second, after thorough rinsing in Tris buffer the PAP procedure for α-smooth muscle actin or Von Willebrand factor visualization was carried out (Kosmehl et al, 1996).

### RESULTS

### Analysis of laminin chain distribution

#### *Normal adult oral squamous epithelium*

In the squamous cell epithelium–stroma interface, a continuous, narrow and sharply contoured BM was demonstrated by collagen type IV immunohistochemistry. An identical BM staining was achieved using antibodies against the laminin chains, α3 (BM165) (Figure 1D), α3 (epiligrin), α5, β1, β3, γ1 and γ2. The α1, α2 and β2 laminin chains were not present in the epithelial BM.

#### *Fetal oral squamous epithelium*

In addition to the laminin chains of adult oral squamous epithelium (α3 (BM165), α3 (epiligrin), α5, β1, β3, γ1, and γ2) the α2 and β2 chain are also expressed (Figure 1A–C). The immunostaining of the BM was continuous and sharply contoured. The α1 chain could not be demonstrated in the epithelial BM.

#### *Hyperplastic and dysplastic oral squamous epithelium*

The laminin chains α3 (BM165), α3 (epiligrin), α5, β1, β3, γ1 and γ2 were detected in the epithelial BM. Compared to normal adult oral squamous epithelium the BM showed irregularities that included thickening and a blurred outline. Additionally, a BM staining for the β2 chain could be demonstrated (Figure 1E). A α2 laminin chain staining was seen in the BM of few pseudopapillas. Occasionally, small segments with a focal loss of laminin chain immunostaining could be observed in dysplastic lesions. The α1 chain could not be demonstrated in the epithelial BM.

#### *Oral squamous cell carcinoma*

The profile of laminin chain expression was similar to that observed in hyperplastic and dysplastic oral squamous epithelium, namely the expression of the laminin α3 (BM165), α3 (epiligrin), α5, β1, β2 (Figure 1F), β3, γ1 and γ2 chains which could be demonstrated around the tumour cell nests. Whereas the α1 chain could not be demonstrated in the BM of the carcinomatous epithelium, an α1 immunostaining was achieved in relation to stromal fibro-/myofibroblasts (Figure 2C). An α2 laminin chain staining could be observed only focally.

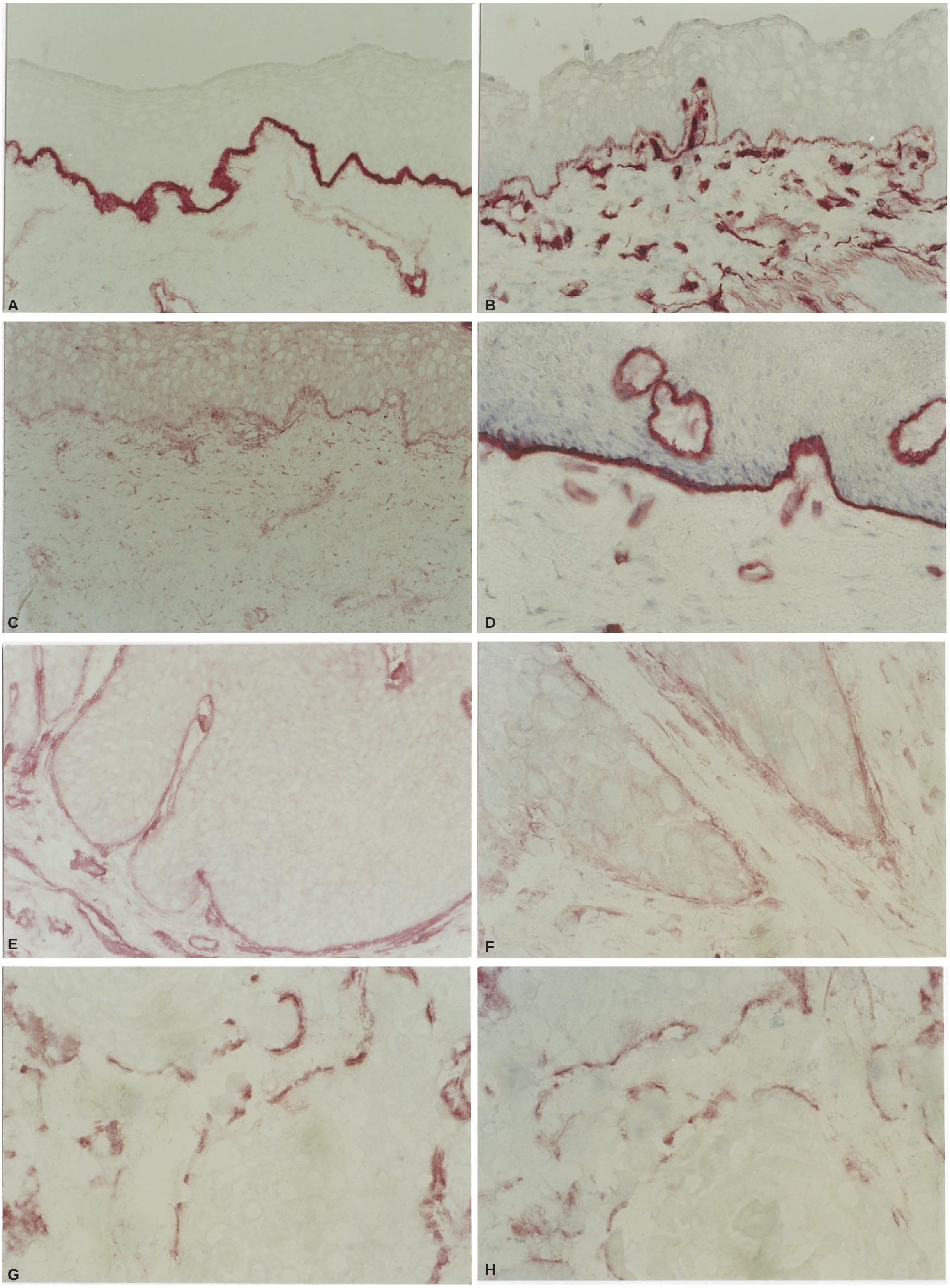

**Figure 1** Fetal oral mucosa with immunohistochemical demonstration of the laminin α3 chain (**A**, BM165), laminin α2 chain (**B**) and laminin β2 chain (**C**) in the squamous epithelial BM region (30th week of gestation, ×150, without counterstaining). In normal adult epithelium the BM represents a sharp contoured line (**D**, laminin α3 chain, BM165, ×150, haematoxylin counterstaining). In hyperplastic epithelium a smooth contoured BM with reexpression of the laminin β2 chain is revealed (**E**, ×150, without counterstaining). The reexpression of the β2 chain is retained in OSCC BM (**F**, ×300, haematoxylin counterstaining). Demonstration of numerous BM breaks in a less differentiated OSCC by immunostaining for the laminin α3 chain (**G**, BM165, ×300, without counterstaining) and the laminin γ2 chain (**H**, ×300, without counterstaining)

© 1999 Cancer Research Campaign

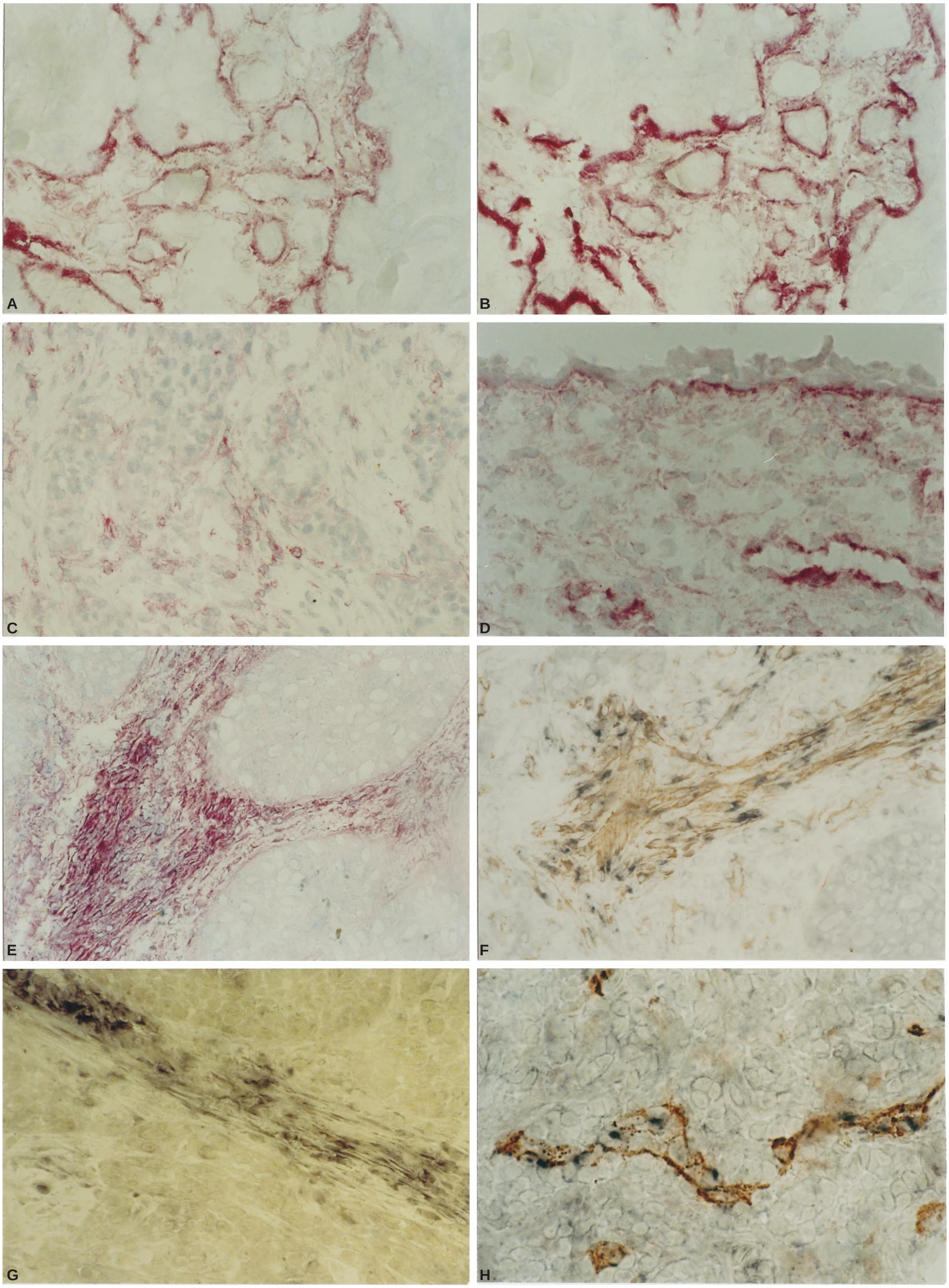

**Figure 2** Demonstration of laminin-5 ($\alpha3\beta3\gamma2$) in the invasion front of a less differentiated OSCC. In addition to the BM staining a focal deposition of the $\alpha3$ chain (**A**, BM165, ×300, without counterstaining) and the $\gamma2$ chain (**B**, ×300, without counterstaining) is shown beneath budding tumour cell complexes. A laminin $\alpha1$ chain staining of the BM is not achieved (**C**, OSCC, ×150, haematoxylin counterstaining). Immunostaining of oncofetal ED-B fibronectin in the BM region as well as diffuse in the stromal compartment of fetal oral mucosa (**D**, 19th week of gestation, clone BC-1, ×300 haematoxylin counterstaining). Strong immunostaining of ED-B fibronectin within the stromal compartment of moderate differentiated OSCC using the recombinant antibody CGS1 (**E**, ×150, haematoxylin counterstaining). OSCC stromal myofibroblasts synthesize ED-B fibronectin as demonstrated by double labelling (ED-B mRNA in situ hybridization, black and $\alpha$-smooth muscle actin immunohistochemistry, brown) (**F**, ×150, without counterstaining). In stromal areas with linear alignment of the fibro-/myofibroblasts (desmoplasia) numerous stromal cells bear the ED-B mRNA in situ hybridization signal (**G**, ×300, without counterstaining). In addition to stromal cells a subset of endothelial cells synthesize ED-B fibronectin as demonstrated by double labelling (**H**, OSCC, ED-B mRNA in situ hybridization, black and factor VIII associated antigen immunohistochemistry, brown, ×300, without counterstaining)

*© 1999 Cancer Research Campaign*

Two further observations were remarkable: first, the immunolabelling of all observed laminin chains was discontinuous, indicating a focal loss of laminin chains in the epithelial BM region of the invasion front (Figure 1G–H, Table 2). The semiquantitatively assessed loss of laminin chains and collagen type IV showed significant differences between G1 and G2/3 carcinomas (α3: $P = 0.0017$, α5: $P = 0.0017$, β1: $P = 0.0032$, γ1: $P = 0.0264$ and collagen type IV: $P = 0.0037$).

Second, an increased staining was achieved for the α3, β3 and γ2 chains in invasive areas. These chains were seen in the cytoplasm of budding tumour cells and outside the BM region in the stroma of the invasion front (Figure 2A–B).

Whereas the antibodies directed against the laminin α3 chain (BM165 and P3H4) extensively stained stromal areas in the invasion front, a comparable but quantitatively lesser β3 and γ2 chain staining was visible in the corresponding stroma of the invasion front. The monoclonal antibody P3H4 also labelled basal lamina structures of stromal myofibroblasts.

### Analysis of oncofetal fibronectin distribution and synthesis

#### *Oncofetal fibronectin immunohistochemistry*

In normal adult and hyperplastic oral mucosa, ED-B fibronectin and de novo glycosylated fibronectin were absent. Embryonal mucosa showed a moderate immunoreactivity for oncofetal fibronectins in the BM region and in the subepithelial stroma (Figure 2D).

In the 13 OSCC ED-B fibronectin (BC1, CGS1) and de novo glycosylated fibronectin (5C10, FDC-6) could be demonstrated by immunohistochemistry. The immunostaining for ED-B fibronectin and de novo glycosylated fibronectin were restricted to the tumour stroma. The immunostaining pattern of both antibodies against ED-B fibronectin was identical, but the staining of the recombinant antibody was slightly more intensive (Figure 2E). Whilst the well-differentiated carcinomas exhibited focal deposits within the stroma and at the invasion front, the moderately/poorly differentiated carcinomas showed a diffuse staining throughout the tumour stroma compartment and at the invasion front. The ED-B fibronectin deposits at the invasion front in well-differentiated carcinomas correlated to the histological aspect of a clumsy invasive carcinoma growth. In addition to the stroma positivity, ED-B fibronectin could also be found immunohistochemically in tumour vessels and in some blood vessels of the pre-existing tissue adjacent to the invasion front (Figure 2H).

#### *ED-B fibronectin RNA/RNA in situ hybridization*

In eight out of 13 carcinomas (two G1 and six G2/3 carcinomas) ED-B fibronectin mRNA could be shown by in situ hybridization. A clear labelling was observed in cells of the stromal compartment (Figure 2F). In G1 carcinomas numerous positive stromal cells with myofibroblastic appearance were identified within the newly formed stroma of verrucous carcinoma projections, in keeping with the immunohistochemical ED-B fibronectin demonstration.

In contrast to the diffuse ED-B fibronectin immunostaining in the G2/G3 carcinomas, the ED-B fibronectin synthesis was limited only to stromal cell foci. The foci were located in newly induced stroma with parallel alignment of myofibroblastic stroma cells (desmoplasia) (Figure 2G). Moreover, a few marked fibroblastic and inflammatory cells could focally be seen at the invasion front. A relation between the number of mRNA containing stromal cells and tumour grade could not be observed. This was also evidenced by statistical analysis. Using double labelling of ED-B fibronectin in situ hybridization and α-smooth muscle actin immunostaining numerous ED-B fibronectin mRNA containing stromal cells were identified as myofibroblasts (Figure 2F).

### DISCUSSION

### Laminin chains in the normal oral squamous epithelium

The laminins represent a continuously growing family of proteins (Kosmehl et al, 1996; Miner et al, 1997), whose members are endowed with different biological functions (Strassburger et al, 1998). Because skin and oral squamous epithelia bear structural and functional resemblances, their similar laminin chain composition is not surprising (Rousselle et al, 1991; Sollberg et al, 1992). According to the visualized chains the following laminin isoforms are possible in the BM of normal oral mucosa: laminin-5, -6 and -10.

Laminin-5 (α3β3γ2) is a typical component of epithelial BMs and is considered the biochemical equivalent of the anchoring filaments fixing basal keratinocytes to the BM. Its significance for the mechanical resistance and protective function of the epithelium has been discussed in the literature (Rousselle et al, 1991, 1995).

The reports in the literature concerning the distribution of the α1 chain in human tissues were based mostly on the application of the antibody 4C7. Recently, however, the monoclonal antibody 4C7 was shown to recognize the laminin α5 chain of laminin-10 (α5β1γ1) and laminin-11 (α5β2γ1) (Tiger et al, 1997). Our data highlight the recently described widespread distribution of laminin-10 in the BMs of various tissue types (Durkin et al, 1997; Miner et al, 1997; Kikkawa et al, 1998).

In contrast to the adult oral squamous epithelium, embryonal oral squamous epithelium showed the presence of the α2 and β2 chains. The following laminin isoforms may be present in the BM of embryonal mucosa: laminin-2, -4, -5, -6, -7, -10 and -11. The α2 chain is mainly associated with the basal lamina of mesenchymal cells (Leivo et al, 1988). More recent studies indicate that the adult epidermal BM contains little (Sollberg et al, 1992; Squarzoni et al, 1997) or no (Hori et al, 1994) laminin α2 chain. Dystroglycan is an important non-integrin receptor for the laminin α2 chain. It is considered crucial for embryonal BM formation (Williamson et al, 1997) and is present in embryonal oral squamous epithelium (unpublished personal observation). Therefore, the expression of the laminin α2 chain in embryonal oral mucosa may be a prerequisite for normal oral morphogenesis.

The α1 chain together with the β1 and γ1 chains forms the prototypic EHS laminin (laminin-1) and is expressed in different BMs (Ryan et al, 1996). Nevertheless, we were unable, however, to detect the laminin α1 chain in embryonal, normal adult, hyper- and dysplastic or carcinomatous epithelial BM using a polyclonal antibody. The specificity of this antibody was checked using normal adult human kidney, with results matching those of Tiger and co-workers (Tiger et al, 1997). Our findings confirm the limited distribution of the laminin α1 chain observed in rodent tissues (Ekblom et al, 1990; Klein et al, 1990).

### Re-expression of the α2 and β2 chains in hyperplastic, dysplastic and carcinomatous oral squamous epithelium

Because the presence of laminin α2 and β2 chains is a regular finding in embryonic tissues (Iivanainen et al, 1994), their

 *© 1999 Cancer Research Campaign*

re-expression in carcinoma indicates a reappearance of embryonic features in BM composition; the following heterotrimeric laminin molecules are possible: laminin-2, -4, -5, -6, -7, -10 and -11.

A re-expression of the β2 chain occurs in vivo not only in OSCC, but also in basal cell carcinoma of the skin (Sollberg et al, 1992), in proliferating benign and malignant lesions of the breast (Kosmehl et al, 1996), and regularly in cultured carcinoma cells (the so-called developmental switch) (Miner et al, 1994; Wewer et al, 1994).

### Structural basement membrane alterations in OSCC demonstrated with antibodies specific for different laminin chains

Basement membrane defects could not be seen at light microscope level in normal or hyperplastic oral squamous epithelium. However, using immunolabelling in hyperplastic lesions a doubling of the width was observed comparable with the findings of Kainulainen et al (1997) in oral lichen planus. In dysplasia and carcinoma BM structural irregularities, including reduplication, splitting, attenuation and breaks (Ueno et al, 1997) have been well documented by electron microscopy. Moreover, immunohistochemical BM defects and a loss of corresponding integrins in head and neck squamous cell carcinomas have been reported (Downer et al, 1993; Harada et al, 1994; Kobayashi et al, 1995; Kosmehl et al, 1995*a*, 1996). In this study, multifocal breaks of BM continuity were observed in the invasion zone of all carcinomas, regardless of the laminin chain specificity of the antibodies. The number and extent correlated well with the malignancy grade. These findings are in line with the results of Harada et al (1994) and Kobayashi et al (1995), who found that an increased loss of BM material (antibodies to EHS-laminin) is associated with a rise in metastatic potential. The semiquantitative evaluation of BM defects reveals differences among the various laminin chains. These differences are not surprising, given that laminin isoform expression is contingent on tissue maturation which varies in the carcinoma invasion front.

The result of laminin α3 chain immunolabelling should be emphasized: first, number and extent of BM defects demonstrated with the α3 chain antibodies (BM165 and P3E4) were fewer and less pronounced than the BM defects demonstrated with antibodies specific to the other laminin chains. Second, in the invasion front there were foci with a strong α3 chain staining outside the original BM region close to budding tumour cells. For the γ2 chain of laminin-5, an invasion-associated increased synthesis was demonstrated in adenocarcinoma of the colon (Pyke et al, 1994; Ueno et al, 1997). In addition, Kainulainen et al (1997) reported a strong deposition of the γ2 chain outside the BM in invasive areas of OSCC. As determined in serial sections, the immunostaining of the α3 chain is more extensive than that of the γ2 chain. The absence of a clear corresponding β1/2 or γ1 chain staining, as well as the demonstrated cytoplasmic accumulation of laminin chains in budding OSCC cells, hint to a disturbed molecule assembly. Comparable results were recently described by Sordat (1998) for colorectal neoplasia.

The focal loss of structural BM organization, and the presence of the γ2 chain and particularly the α3 laminin chain on tumour cell surface and in the stroma adjacent to invasive carcinoma cell clusters, may support the tumour cell invasion (Kosmehl et al, 1996). Invasion mediated via newly deposited laminin-5 containing BM has previously been suggested in pancreatic adenocarcinoma (Tani et al, 1997). In case of OSCC cells the invasion related laminin-5 immunostaining is not associated with an organized BM as shown in vitro (Berndt et al, 1997). Whilst the role of laminin-5 as a strong adhesive for keratinocytes in vivo and in vitro is generally accepted (Rousselle et al, 1991), the ability to promote keratinocyte migration is still controversial (O'Toole et al, 1997). Nevertheless, the majority of experiments suggest that it can promote cell motility (Zhang et al, 1996; Tani et al, 1997).

### ED-B fibronectin synthesis indicates tumour stroma recruitment

Our results show that, except for some highly differentiated carcinomas, oncofetal fibronectins (ED-B fibronectin, de novo glycosylated fibronectin) permeate the whole stromal compartment of OSCC (cp. Mandel et al, 1994). In contrast to OSCC, breast and colon carcinomas display only focal oncofetal fibronectin deposits if at all (Kaczmarek et al, 1994; Pujuguet et al, 1996). As previously described, newly formed vessels within tumour stroma contain ED-B fibronectin (Castellani et al, 1994). We have found that some, possibly growth factor-stimulated, blood vessels neighbouring the invasion front are also ED-B fibronectin-positive. The new phage antibody CGS1 (Carnemolla et al, 1996) generates comparable staining patterns to the mouse monoclonal antibody BC1, proving to be a valuable and easy to handle immunohistochemical tool. The in situ hybridization signal for ED-B fibronectin was confined to focal accumulations of spindle-shaped stromal cells with abundant cytoplasm. Based on their immunocytochemical findings in stroma cell cultures of breast carcinomas, Brouty-Boyé and Magnien (1994) suggested that the stromal myofibroblasts were the source of ED-B fibronectin in malignant tumours. Indeed, the ED-B fibronectin in situ hybridization labelled stromal cells show myofibroblastic features by light microscopy. As previously demonstrated also in palmar fibromatosis (Dupuytren's disease), myofibroblasts abundantly synthesize ED-B fibronectin (Kosmehl et al, 1995*b*).

A correlation of the number of ED-B fibronectin-positive stromal cells to malignancy grade could not be detected. The focal accumulations of ED-B fibronectin synthesizing cells were found in the stromal compartment of papillary projections of highly differentiated carcinomas (area of stroma formation), or in stromal areas with a parallel alignment of fibro-/myofibroblasts (desmoplasia). Therefore, ED-B fibronectin synthesis may indicate stroma recruitment inclusive of active desmoplastic stroma reaction. The high ED-B fibronectin synthesis during the formation of an aligned fibrocollagenous stroma concurs with the observation of Nickeleit et al (1995), who found the strongest ED-B fibronectin in situ hybridization signal during the sclerosis stage of experimental autoimmune glomerulonephritis in rats. Moreover, single spindle-shaped cells and groups of inflammatory cells positive for ED-B fibronectin synthesis occur regularly at the invasion front. An expression of ED-B fibronectin in macrophages has already been described in experimental glomerulonephritis and granulation tissue (Barnes et al, 1995). Because ED-B fibronectin enhances adhesion and spreading of several cell types in vitro (Chen and Culp, 1996; Hashimoto-Uoshima et al, 1997), the ED-B fibronectin synthesis at the invasion front of OSCC should be discussed in relation to an altered cellular adhesion, an advancement of cellular migration and formation of an invasive phenotype (Kosmehl et al, 1996).

*© 1999 Cancer Research Campaign*

### Embryonic extracellular matrix features in OSCC

The re-expression of a fetal differentiation pattern in malignant tumours (so called retrodifferentiation) has been known for nearly 2 decades (Uriel et al, 1979). The embryonic phenotype in malignant tumours entails not only the tumour cells themselves, but also the extracellular matrix pattern: re-expression of ED-B fibronectin, de novo glycosylated fibronectin and the laminins-2,-4, -7 and 11. Whereas laminin synthesis is attributed to the epithelial (i.e. carcinoma) cells (Pyke et al, 1994; Ueno et al, 1997), the oncofetal ED-B fibronectin matrix is generated by recruited stromal cells (myofibroblasts), as evinced in this study. The alternative splicing of fibronectin and the expression of laminin-5 chains by keratinocytes are regulated by the growth factor TGFβ (Borsi et al, 1990; Berndt et al, 1995; Korang et al, 1995). As a consequence, a co-ordinated extracellular matrix remodelling in the carcinoma invasion front may be suggested.

### ACKNOWLEDGEMENTS

The authors are grateful to Professor Luciano Zardi from Genova for encouragement and critical advice in the ECM studies. The authors thank Dr. Thomas Wiley from the Department of International Affairs; National Institute for Cancer Research, Genova for critical reading of the manuscript. We thank Mrs Christiane Rudolph and Mrs Carola König for skilful technical assistance. The investigation was supported by the grant ThMWK no. 973117.

### REFERENCES

Barnes JL, Torres E, Mitchell RJ and Peters JH (1995) Expression of alternatively spliced fibronectin variants during remodelling in proliferative glomerulonephritis. *Am J Pathol* **147**: 1361–1371

Berndt A, Hyckel P, Könnecker A, Katenkamp D and Kosmehl H (1997) Oral squamous cell carcinoma invasion is associated with a laminin-5 matrix re-organisation but independent of basement membrane and hemidesmosome formation. Clues from an in vitro invasion model. *Inv Metast* **17**: 251–258

Berndt A, Kosmehl H, Mandel U, Gabler U, Luo X, Celeda D, Zardi L and Katenkamp D (1995) TGFβ and bFGF synthesis and localization in Dupuytren’s disease (nodular palmar fibromatosis) relative to cellular activity, myofibroblast phenotype and oncofetal variants of fibronectin. *Histochem J* **27**: 1041–1020

Borsi L, Carnemolla B, Castellani P, Rosellini C, Vecchio D, Allemani G, Chang SE, Taylor-Papadimitriou, Pande H and Zardi L (1987) Monoclonal antibodies in the analysis of fibronectin isoforms generated by alternative splicing of mRNA precursors in normal and transformed human cells. *J Cell Biol* **104**: 595–600

Borsi L, Castellani P, Risso AM, Leprini A and Zardi L (1990) Transforming growth factor-β regulates the splicing pattern of fibronectin messenger RNA precursor. FEBS **261**: 175–178

Brouty-Boye D and Magnien V (1994) Myofibroblast and concurrent ED-B fibronectin phenotype in human stromal cells cultured from non-malignant and malignant breast tissue. *Eur J Cancer* **30A**: 66–73

Bryne M, Koppang HS, Lilleng R and Kjaerheim A (1992) Malignancy grading of the deep invasive margins of oral squamous cell carcinoma has high prognostic value. *J Pathol* **166**: 375–381

Burton-Wurster N, Lust G and Macleod JN (1997) Cartilage fibronectin isoforms: in search of functions for a special population of matrix glycoproteins. *Matrix Biol* **15**: 441–454

Carnemolla B, Balza E, Siri A, Zardi L, Nicotra MR, Bigotti A and Natali PG (1989) A tumor-associated fibronectin isoform generated by alternative splicing of messenger RNA precursors. *J Cell Biol* **108**: 1139–1148

Carnemolla B, Neri D, Castellani P, Leprini A, Neri G, Pini A, Winter G and Zardi L (1996) Phage antibodies with pan-species recognition of the oncofetal angiogenesis marker fibronectin ED-B domain. *Int J Cancer* **68**: 397–405

Castellani P, Viale G, Dorcaratto A, Nicolo G, Kaczmarek J, Querze G and Zardi L (1994) The fibronectin isoform containing the ED-B oncofetal domain: a marker of angiogenesis. *Int J Cancer* **59**: 612–618

Chen W and Culp LA (1996) Adhesion mediated by fibronectin’s alternatively spliced Edb (EIIIB) and its neighbouring type III repeats. *Exp Cell Res* **223**: 9–19

Downer CS, Watt FM and Speight PM (1993) Loss of α6 and β4 integrin subunits coincides with loss of basement membrane components in oral squamous cell carcinomas. *J Pathol* **171**: 183–190

Durkin ME, Loechel F, Mattei MG, Gilpin BJ, Albrechtsen R and Wewer UM (1997) Tissue-specific expression of the human laminin alpha5-chain, and mapping of the gene to human chromosome 20q13.2–13.3 and to distal mouse chromosome 2 near the locus for the ragged (Ra) mutation. *FEBS Lett* **411**: 296–300

Ekblom M, Klein G, Mugrauer G, Fecker L, Deutzmann R, Timpl R and Ekblom P (1990) Transient and locally restricted expression of laminin A chain mRNA by developing epithelial cells during kidney organogenesis. *Cell* **60**: 337–346

Engvall E, Earwicker D, Haaparanta T, Ruoslahti E and Sanes JR (1990) Distribution and isolation of four laminin variants; tissue restricted distribution of heterotrimers assembled from five different subunits. *Cell Regul* **1**: 731–740

Harada T, Shinohara M, Nakamura S and Oka M (1994) An immunohistochemical study of the extracellular matrix in oral squamous cell carcinoma and its association with invasive and metastatic potential. *Virchows Arch* **424**: 257–266

Hashimoto-Uoshima M, Yan YZ, Schneider G and Aukhil I (1997) The alternative spliced domains EIIIB and EIIIA of human fibronectin affect cell adhesion and spreading. *J Cell Sci* **110**: 2271–2280

Hori H, Kanamori T, Mizuta T, Yamaguchi N, Liu Y and Nagai Y (1994) Human laminin M chain: epitope analysis of its monoclonal antibodies by immunoscreening of cDNA clones and tissue expression. *J Biochem* **116**: 1212–1219

Hruban RH, Kuhajda FP and Mann RB (1987) Acute myelofibrosis. Immunohistochemical study of four cases and comparison with acute megakaryocytic leukemia. *Am J Clin Pathol* **88**: 578–588

Hsi BL and Yeh C-JG (1986) Monoclonal antibodies to human amnion. *J Reprod Immunol* **9**: 11–21

Iivanainen A, Vuolteenaho R, Sainio K, Eddy R, Shows TB, Sariola H and Tryggvason K (1994) The human laminin β2 chain (s-laminin): structure, expression in fetal tissues and chromosomal assignment of the LAMB2 gene. *Matrix Biol* **14**: 489–497

Kaczmarek J, Castellani P, Nicolo G, Spina B, Allemanni G and Zardi L (1994) Distribution of oncofetal fibronectin isoforms in normal, hyperplastic and neoplastic human breast tissues. *Int J Cancer* **58**: 11–16

Kainulainen T, Autio-Harmainen H, Oikarinen A, Salo S, Tryggvason K and Salo T (1997) Altered distribution and synthesis of laminin-5 (Kalinin) in oral lichen planus, epithelial dysplasias and squamous cell carcinomas. *Br J Dermatol* **136**: 331–336

Kikkawa Y, Sanzen N and Sekiguchi K (1998) Isolation and characterization of laminin-10/11 secreted by human lung carcinoma cells. Laminin-10/11 mediates cell adhesion through integrin alpha3 beta1. *J Biol Chem* **273**: 15854–15859

Klein G, Ekblom M, Fecker L, Timpl R and Ekblom P (1990) Differential expression of laminin A and B chains during development of embryonic mouse organs. *Development* **110**: 823–837

Kobayashi Y, Nakajima T and Saku T (1995) Loss of basement membranes in the invading front of O-1N, hamster squamous cell carcinoma with high potential of lymph node metastasis: An immunohistochemical study for laminin and type IV collagen. *Pathol Int* **45**: 327–334

Korang K, Christiano AM, Uitto J and Mauviel A (1995) Differential cytokine modulation of the genes LAMA3, LAMB3, and LAMC2, encoding the constitutive polypeptides, α3, β3, and γ2, of human laminin 5 in epidermal keratinocytes. *FEBS Lett* **368**: 556–558

Kornblihtt AR, Pesce CG, Alonso CR, Cramer P, Srebrow A, Werbajh S and Muro AF (1996) The fibronectin gene as a model for splicing and transcription studies. *FASEB J* **10**: 248–257

Kosmehl H, Berndt A, Katenkamp D, Hyckel P, Stiller KD, Gabler U, Langbein L and Reh T (1995*a*) Integrin receptors and their relationship to cellular proliferation and differentiation of oral squamous cell carcinoma. A quantitative immunohistochemical study. *J Oral Pathol Med* **24**: 343–348

Kosmehl H, Berndt A, Katenkamp D, Mandel U, Bohle R, Gabler U and Celeda D (1995*b*) Differential expression of fibronectin splice variants, oncofetal glycosylated fibronectin and laminin isoforms in nodular palmar fibromatosis. *Path Res Pract* **191**: 1105–1113

Kosmehl H, Berndt A and Katenkamp D (1996) Molecular variants of fibronectin and laminin: structure, physiological occurrence and histopathological aspects. *Virchows Arch* **429**: 311–322

 *© 1999 Cancer Research Campaign*

Leivo I and Engvall E (1988) Merosin, a protein specific for basement membranes of Schwann cells, striated muscle, and trophoblast, is expressed late in nerve and muscle development. *Proc Natl Acad Sci USA* **85**: 1544–1548

Loridon-Rosa B, Vielh P, Matsuura H, Clausen H, Cuadrado C and Burtin P (1990) Distribution of oncofetal fibronectin in human mammary tumors: immunofluorescence study on histological sections. *Cancer Res* **50**: 1608–1612

Mandel U, Therkildsen MH, Reibel J, Sweeney, Matsuura H, Hakamori S-I, Dabelsteen E and Clausenn H (1992) Cancer-associated changes in glxcosylation of fibronectin. Immunohistological localization of oncofetal fibronectin defined by monoclonal antibodies. *APMIS* **100**: 817–826

Mandel U, Gaggero B, Reibel J, Therkildsen MH, Dabelsteen E and Clausen H (1994) Oncofetal fibronectins in oral carcinomas: correlation of two different types. *APMIS* **102**: 695–702

Marinkovich PM, Lunstrum GP, Keene DR and Burgeson RE (1992) The dermal-epidermal junction of human skin contains a novel laminin variant. *J Cell Biol* **119**: 695–703

Matsuura H and Hakamori S (1985) The oncofetal domain of fibronectin defined by monoclonal antibody FDC-6: its presence in fibronectins from fetal and tumor tissues and its absence in those from normal adult tissues and plasma. *Proc Natl Acad Sci USA* **82**: 6517–6521

Miner JH and Sanes JR (1994) Collagen IV α3, α4 and α5 chains in rodent basal laminae: sequence, distribution, association with laminins, and development switches. *J Cell Biol* **127**: 879–891

Miner JH, Patton BL, Lentz SI, Gilbert DJ, Snider WD, Jenkins NA, Copeland NG and Sanes J (1997) The laminin α chains: expression, developmental transitions, and chromosomal locations of α1–5, identification of heterotrimeric laminins 8–11, and cloning of a novel α3 isoform. *J Cell Bio* **137**: 685–701

Nickeleit V, Zagachin L, Nishikawa K, Peters JH, Hynes RO and Colvin RB (1995) Embryonic fibronectin isoforms are synthesised in crescents in experimental autoimmune glomerulonephritis. *Am J Pathol* **147**: 965–978

Odermatt BF, Lang AB, Rüttner JR, Winterhalter KH and Trüeb B (1984) Monoclonal antibodies to human type IV collagen: useful reagents to demonstrate the heterotrimeric nature of the molecule. *Proc Natl Acad Sci USA* **81**: 7343–7347

O'Toole EA, Marinkovich MP, Hoeffler WK, Furthmayr H and Woodley DT (1997) Laminin-5 inhibits human keratinocyte migration. *Exp Cell Res* **233**: 330–339

Pujuguet P, Hammann A, Moutet M, Samuel JL, Martin F and Martin M (1996) Expression of fibronectin ED-$A^+$ and ED-$B^+$ isoforms by human and experimental colorectal cancer. *Am J Pathol* **148**: 579–592

Pyke C, Roemer J, Kallunki P, Lund LR, Ralfkiaer E, Danoe K and Tryggvason K (1994) The γ2 chain of kalinin/laminin-5 is preferentially expressed in invading malignant cells in human cancers. *Am J Pathol* **145**: 782–791

Rousselle P, Lunstrum GP, Keene DR and Burgeson RE (1991) Kalinin: an epithelium-specific basement membrane adhesion molecule that is a component of anchoring filaments. *J Cell Biol* **114**: 567–576

Rousselle P, Golbik R, van-der-Rest M and Aumailley M (1995) Structural requirement for cell adhesion to kalinin (laminin-5). *J Biol Chem* **270**: 13766–13770

Ryan M, Christiano AM, Engvall E, Wewer U, Miner JH, Sanes JR and Burgeson RE (1996) The functions of laminins: lessons from in vivo studies. *Matrix Biol* **15**: 369–381

Sanes JR and Chiu AY (1983) The basal lamina of the neuromuscular junction. Cold *Spring Harbor Symp Quant Biol* **48**: 776–678

Skalli O, Ropraz P, Trzeciak A, Benzonana G, Gillesen D and Gabbiani G (1986) A monoclonal antibody against alpha-smooth muscle actin: a new probe for smooth muscle differentiation. *J Cell Biol* **103**: 2787–2796

Sollberg S, Peltonen J and Uitto J (1992) Differential expression of laminin isoforms and β4 integrin epitopes in the basement membrane zone of normal human skin and basal cell carcinomas. *J Invest Dermatol* **98**: 864–970

Sordat I, Bosman FT, Dorta G, Rousselle P, Aberdam D, Blum AL and Sordat B (1998) Differential expression of laminin-5 subunits and integrin receptors in human colorectal neoplasia. *J Pathol* **185**: 44–52

Squarzoni S, Villanova M, Sabatelli P, Malandrini A, Toti P, Pini A, Merlini L, Guazzi GC and Maraldi NM (1997) Intracellular detection of the laminin α2 chain in skin by electron microscopy immunocytochemistry: comparison between normal and laminin α2 chain deficient subjects. *Neuromusc Disord* **7**: 91–98

Strassburger S, Berndt A, Hyckel P, Katenkamp D and Kosmehl H (1998) Differential expression of laminin chains in the human major salivary gland. *Histochem J* **30**: 81–88

Tani T, Lumme A, Linnala A, Kivilaakso E, Kiviluoto T, Burgeson RE, Kangas L, Leivo I and Virtanen I (1997) Pancreatic carcinomas deposit laminin-5, preferably adhere to laminin-5, and migrate on the newly deposited basement membrane. *Am J Pathol* **151**: 1289–1302

Thorup AK, Dabelsteen E, Schou S, Gil SG, Carter WG and Reibel J (1997) Differential expression of integrins and laminin-5 in normal oral epithelia. *APMIS* **105**: 519–530

Tiger CF, Champliaud MF, Pedrosa-Domellof F, Thornell LE, Ekblom P and Gullberg D (1997) Presence of laminin alpha 5 chain and lack of laminin alpha 1 chain during human muscle development and in muscular dystrophies. *J Biol Chem* **272(45)**: 28590–28595

Ueno S, Osugi Y, Nishimura A, Shinoda Y, Mushimoto K and Shirasu R (1997) Ultrastructural localization of collagen type IV and laminin expression in the epithelial basement membrane of oral carcinomas. *Med Electron Microsc* **30**: 37–42

Uriel J (1979) Retrodifferentiation and the fetal patterns of the gene expression in cancer. *Adv Canc Res* **29**: 127–174

Wayner EA, Gil SG, Murphy GF, Mark SW and Carter WG (1993) Epiligrin, a component of epithelial basement membranes is an adhesive ligand for α3β1 positive T lymphocytes. *J Cell Biol* **121**: 1141–1152

Wewer U, Albrechtsen R, Manthorpe M, Varon S, Engvall E and Ruoslahti E (1983) Human laminin isolated in a nearly intact, biologically active form from placenta by limited proteolysis. *J Biol Chem* **258**: 12654–12660

Wewer U, Wayner EA, Hoffstrom BG, Lan F, Mayer-Nielsen B, Engvall E and Albrechtsen R (1994) Selective assembly of laminin variants by human carcinoma cells. *Lab Invest* **71**: 719–730

Williamson RA, Henry MD, Daniels KJ, Hrstka RF, Lee JC, Sunada Y, Ibraghimov-Beskrovnaya O and Campbell KP (1997) Dystroglycan is essential for early embryonic development: disruption of Reichert's membrane in Dag-1 null mice. *Hum Mol Genet* **6**: 831–841

Xia P and Culp LA (1995) Adhesion activity in fibronectin's alternatively spliced domain Eda (EIIIA): complementary to plasma fibronectin functions. *Exp Cell Res* **217**: 517–527

Zardi L, Carnemolla B, Siri A, Santi L and Accolla RS (1980) Somatic cell hybrids producing antibodies specific to human fibronectin. *Int J Cancer* **25**: 325–329

Zhang K and Kramer RH (1996) Laminin-5 deposition promotes keratinocyte motility. *Exp Cell Res* **227**: 309–322

© 1999 Cancer Research Campaign
